# Processualizing Data: Variants of Process‐Produced Data

**DOI:** 10.1111/cars.12280

**Published:** 2020-05-17

**Authors:** Tilo Grenz

**Affiliations:** ^1^ University of Vienna

## Abstract

The greatly increased importance of diachronic process perspectives in the social sciences has led to process‐produced data (PPD) becoming one of the main topics of this debate. However, its current use is peculiarly ambivalent. It oscillates between substantialist understandings and self‐evident use. This article stresses the beforehand conceptual decisions made by the researcher that eventually define data, that is, which materials become PPD. Because process‐oriented research rests on heterogenous conceptions of temporality, it is especially these conceptions researchers must make transparent. Drawing on social constructivism, extended by path dependency, happenings, and events, the article exemplifies one conceptual foundation and, using insights from two research projects (historical discussion circles and trajectories of digital infrastructures), a particular specification of PPD. The article thus contributes to the necessary methodological reflection on PPD, and, at the same time, responds to the need for diachronic social research in order to grasp contemporary processes of digitalization.

## Résumé

L'importance grandement accrue des perspectives du processus diachronique dans les sciences sociales a mené à des données produites par les processus qui deviennent l'un des principaux thèmes de ce débat. Cependant, son utilisation actuelle est particulièrement ambivalente. Elle oscille entre des compréhensions substantialistes et l'utilisation évidente qui va de soi. Cet article souligne à l'avance les décisions conceptuelles prises par le chercheur qui définissent éventuellement les données, c.‐à‐d. quel matériel devient des données produites par les processus. Étant donné que la recherche axée sur les processus repose sur des conceptions hétérogènes de temporalité, ce sont surtout ces conceptions que les chercheurs doivent rendre transparentes. Tirant parti du constructivisme social, élargi par la dépendance de l'itinéraire, les happenings et les événements, cet article exemplifie un fondement conceptuel et, à l'aide des perspectives de deux projets de recherche (cercles de discussion historique et trajectoires des infrastructures numériques), une caractéristique particulière des données produites par les processus. Cet article contribue donc à la réflexion méthodologique demandée sur les données produites par les processus, et répond à la fois au besoin de recherche sociale diachronique afin de saisir les processus contemporains de numérisation.

## TEMPORALITY OF PROCESSES, PROCESS‐PRODUCED AND DIGITAL DATA

OVER THE PAST YEARS, empirical social research has increasingly called to supplement a synchronous with a diachronous research perspective. The demand goes back decisively to the rediscovery of historical approaches in and for sociology and to attempts to construct not only a fruitful combination of but also the integration of sociology and history (already Abbott 2001; Baur 2011; Sewell 1996). This move brought temporality and processes unfolding in time on center stage (Abbott 2016). On the one hand, there is broad agreement on what is meant by synchronous research perspectives. Referring to a prominent perspective, empirical‐material cultural analysis is the research that fixes a certain point in time for the interpretation of phenomena in order to uncover their internal logic and coherence in an interpretative way: “Every cultural analysis necessarily entails a synchronic moment of this sort (…)” (Sewell 2005:178; see also Bryman 2004:281; criticizing statics cf. Lachmann 2013:4). On the other hand, there is an extraordinary variety on what “diachronic” means and how empirical diachronic research can be carried out. Within Historical Social Sciences, the vote for “diachronic social sciences” (Best 2008, own translation), “capable of giving sociological empirics the necessary temporal depth” (Best 1988:12, own translation) became prominent. However, the expressiveness of this statement masks the fact that the term “diachronic” implies deep‐seated epistemological differences, which are methodologically significant.

This significance can be shown in the temporal conception of diachronicity: There are, as it were, “hard” positivistic understandings of time series, for example, which correspond to the idea of the “temporality of social events as a duration or a continuation in the vivid manner of a diagram” (Schwietring 2015:152, own translation). The basic structure corresponds to a deductive orientation—the hypothesis‐led search for causal explanations in respective data sets—which is consequently reserved for this strand of diachronic social research. On the other hand, there are interpretative‐reconstructive understandings in which “what happens […] occurs only apparently in chronological order” and rather always only as a realization, as a (new) creation in a “here and now.” Either timeless, in the water of the “ahistorical or synchronic pretense of anthropology” (Hastrup 1990:45), as already mentioned, or methodologically explicitly reflected, the cultural contingency of time is on the agenda. The analyses of Historical Social Sciences that are related to time points and time series, which were positioned as decidedly quantitative “diachronic social science” (Best 2008:74; cf. Clubb 1980), are thus contrasted with interpretative analyses, based on Weberian “understanding sociology.” Synchronic social research that rests on temporal narratives serves as integrative fundament of such a diachronic perspective (Abbott 2016:77 ff.). For instance, in biographical research, the “diachronic time structure” unfolds in the narrated life story and thus, in principle, as “represented time”—in contrary to causal‐transitory time analyses (Dausien 2018:203, own translation).

Approaches that have been assigned to the *New* Historical Sociology (see the pointed overview by Schützeichel 2009) explicitly deal with the distinction between deductive and inductive implied therein. Abbott (1992:64) in his discussion of case construction identifies an ontological difference between “cases in standard quantitative methods” and those in “historical case studies” (p. 53). In order to label the difference, he distinguishes “population/analytic” and “case/narrative” studies, which echoes the mentioned distinction within diachronic perspectives. Baur (2011:1233 ff.), on the other hand, explicitly avoids this distinction. She regards historical‐sociological case studies as a third way of empirical social research that can hardly be assigned to one or another fraction (Baur 2011; cf. also Schützeichel 2015:125). Baur (2011) therefore demands mixed methods designs, in order to bridge the inadequate dichotomy of “quantitative” and “qualitative” data.

In this perspective, which emphasizes the diachronic orientation and the plurality of data sources, a certain kind of data that is referred to as “process‐produced data” (PPD) has become key (Bauernschmid 2009; Baur 2011:1234 ff.; Clubb and Scheuch 1980). Interestingly enough, the current and broad use of PPD is peculiarly ambivalent. It oscillates between a substantialist understanding and its self‐evident use. Introduced as a fundamental element of everyday life by Rokkan (1969a, 1969b) in the 1960s, PPD's use in social sciences eventually experienced a systematic constriction especially on standardized administrative data (e.g., Müller 1977; cf. the extensive collection in Best 2008; Clubb and Scheuch 1980; Schröder 2006) that is currently being widened up again (Bauernschmid 2009).

Rokkan (cf. Bauernschmidt 2009:214) introduced “PPD” as part of his discussion of the increasing relevance of (machine) data archives for social sciences. Referring to the “archival revolution,” Rokkan (1969a:63) differentiated this kind of data from data produced by official “bookkeeping” agencies and, even more, from data elicited by researchers. Ranging from lower to higher “Degree[s] of Structuration,” Rokkan (1976:454) enumerated various kinds of materials such as “graffiti, posters, pamphlets, newspaper stories, private diaries, letters, parliamentary debates, public ceremonies.” PPD thus are in a maximum contrast to research‐generated data; they are generated through the very processes of “living, working, interacting in the societies […]—from plain material evidence through all kinds of artifacts to the varieties of symbolic representations of ideas, activities, and events, whether drawings, tales, messages, or documents” (Rokkan 1969b:4). Although having a focus on “official” documentation and archiving services, the broad material range and definition accentuate that individuals and everyday action generate this kind of data; that is, such data are not produced for research purposes, but left behind as a by‐product of everyday life. In this respect, PPD connects to the epistemological reflections on traces and their epistemological ambivalence between evidence and interpretation (foundationally Ginzburg 1979; Grenz 2017:122–24; Krämer 2007). Consequently, similar to traces, PPD indicate processes because they are not created with communicative intention.

This “quality” connects PPD to the recent “data debate” in digital sociology (Caliandro and Gandini 2017; Marres 2017). Two connecting strands of this debate are of particular importance here. The first particularly concerns critiques that address the taken‐for‐grantedness of digital data as carriers of “objective reality” (Crawford, Miltner, and Gray 2014; Reigeluth 2014)—that “data is being icily naturalized, with its institutional and methodological preconditions being marginalized from discussion” (Davies, para. 7; quoted by Crawford et al. 2014:1667). Emphasizing the contextuality of data, recent reflections on *digital* traces present an important counter‐proposal (Hepp, Breiter, and Friemel 2018:442) and, thus, that data are always produced. Second, and directly related, within digital methods the (uncontrollable) origins of data or traces and the specificity of certain digital environments (like Twitter) have been emphasized (Gangneux 2019; Marres and Weltverde 2013). Contrary to this intensive preoccupation with the “origin” of data, the methodological question of what researchers (on the background of certain theoretical assumptions) define as PPD has not been discussed. Answers become even more important since sociology of the digital discovers the importance of process perspectives as access to fast‐moving digital architectures and sociomaterial negotiations (Brügger and Finnemann 2013; Bruns and Burgess 2016; Grenz and Kirschner 2018; Snee et al. 2016; Venturini and Latour 2010; cf. Section “Processualizing PPD: Conceptual Foundations of PPD and Two Examples”).

Consequently, this article takes up the demand for a further methodological reflection on PPD as complex data (Baur 2011:1234 f.; see Section “Processualizing PPD: Conceptual Foundations of PPD and Two Examples”). While in the current debate questions of its origin, its purposes (e.g., as kind of secondary data) and its classification dominate, the article stresses the often‐times overlooked entanglement of process perspectives (i.e., theories on the processual nature of the social) and embedded concepts of temporality. A full‐blown methodological conceptualization of PPD for process‐oriented research needs to consider respective process‐theoretical underpinnings. It is these (often times implicit) assumptions and decisions that “make” certain kinds of material expressions PPD; and it is this particular “move” to theory guiding empirics (Eisewicht and Grenz 2018; Kelle 2014; Meinefeld 2004) that stands against the openness and ubiquity of PPD, which has understandably been problematized (Baur 2011:1234 f.). In empirical research, data, be it researcher elicited or “raw” PPD, is never simply “collected” but produced (McNeill and Chapman 2005:183). Regarding process‐oriented research, this results in the necessity of a strict processualization of PPD, according to the thesis developed here. Since this processualization must be done on the basis of temporal perspectives, for which in turn different understandings exist, this article starts with the composition and presentation of a process‐oriented frame of reference.

Therefore, in the following section, three different perspectives for the temporality of processes will be identified and described (differential, sequential, and consequential). Drawing on the consequential perspective, particular theoretical and conceptual presuppositions will be made transparent in order to specify PPD. Thereupon, the subsequent section refers to the methodological debate on the relationship between theory and empirics and finally presents two temporal perspectives (happening frame and the event frame). This distinction, consequently, guides certain specifications of PPD and connected goals. In order to substantiate and illustrate the differentiation, examples from own research projects will be used: the first example comes from a history of sociology project on Viennese discussion circles of the 1920s and 1930s; the second example draws on insights on the dynamics of digital infrastructures (i.e., trajectories of digital social worlds). The final section concludes and discusses the contribution in regard to the necessity of processualizing PPD.

## TEMPORALITY OF PROCESSES: COMPOSING ELEMENTS OF DEFINING PPD

In sociological theory in general and in the discussion of process theories in particular, various “classical” temporalities of the emergence of social reality have been distinguished (Abbott 2001; Sewell 1996 [1990]). This article takes this important debate into account but starts with the decidedly methodological implications: What kind of processes are (currently) coming into view and what relevance does time and temporality have for the development of culture and society? Three widely established perspectives can be identified, which differently start from a temporality of the social.

The first perspective, which is to be described here as *differential*, understands time as a phenomenon as it is produced, stabilized and enforced differentially in various sociocultural contexts. In this context, time is emphasized as “social time” and thus corresponds to the plurality of cultures. The self‐evident ways in which, for instance, tempo (in plural) is internalized come to the fore when they get into conflict with one another. Time, as the cultural dimension of the social, for which, for example, different speeds of movement are regarded as an indicator of the different “pace of life” worldwide, takes the form of “temporal norms” (Levine 2006:164). Here, time is the subject of cultural analysis and not the (temporal) integral of the emergence and change of culture and society as emphasized by process‐oriented research. Cultural actions are here, to put it in a nutshell, frozen in time—expressly considered in synchronous comparison.

The *sequential perspective*, second, also follows a culturalist framing. However, it rests on a fundamental processuality of the social. Social order is the consequence and at the same time the starting point of a constant (re)production of the social (cf. Rawls 2005). In mostly repetitive practices (processes), moments of “creativity” and change arise. This perspective refers to procedures that allow us to identify and describe conduct, which is understood as a process in principle, in the details of its recurring course or execution. Culture is always culture in action, or more precisely culture in temporal conducts of action. Time here is a solid, continuous, momentum insofar as practices have their place in timely sequence (Reckwitz 2002:255). Even if there are varieties of practice theory, it is remarkable that temporality here can be ontologized in a particular way, because, by definition, it is a matter of temporally condensed sequencing and, thus, practices get by without precedence (cf. Scheffer 2019 for a further development). Processes, thus, are always situated processes with order emerging sequentially. History by means of precedents or paths does not play an integral role.

The third perspective, which is particularly compatible with the logic of the New Historical Sociology, will be taken up and developed in this article. The *consecutive perspective* understands reality as something that only historically has developed (and still, continually, is developing; cf. Abbott 2016). Reality only builds up in the diachronic course. In the passing of time, in a “before‐after‐then”, social “factuality” (objectivations) emerges. Social constructivism is a key representative of this perspective. Similar to the first perspective, this one considers the coercive character of different social time structures as institutional phenomena (Berger and Luckmann 1991:40). However, there is a difference in perspective to the effect that knowledge and action appear to be historical in principle—bound by space and time and, not arbitrarily, developed in the course of human history (Berger and Luckmann 1991:65 ff.). Thus, what follows is a nonsituative understanding of the social as a process that is linked to the production, preservation and transmission of knowledge (Abbott 2001; Endress 2018; Pfadenhauer and Grenz 2020). Although action is sequential, similar to the way described in the second perspective, it is “socially channeled” (Endress 2018:58) against the background of socially derived, historically constructed knowledge.

Temporality in the consecutive perspective thus does not primarily focus on the “constant” production of the social, which made it possible to refrain from the consequences of prior action or even to ignore precedence. The consecutive perspective accentuates the sometimes more, sometimes less institutionalized ways of thinking, acting and working in the course of time; that is, in a historical sequence of action and products of (preceding) action (Knoblauch 2018). Action and any products of action not only always have a sociohistorical signature but are also always an expression of a position within and between periods of history. However, these processes are neither teleological—characterized by specific directionality—nor are they the result of any a priori mechanisms (cf. the differentiation by Sewell 1996). They are the result of the sociohistorical processes anchored in and over time. In this perspective, time is an integral of the emergence of reality. Albeit older, social constructivism thus provides an integrative framework (Endress 2018) that is not only an expression of the recognition of the fundamental historicity of social reality, but also consistently pursues the program of a “so and not otherwise” becoming of reality (Weber 1988:257, own translation).

The work carried out in the more recent field of New Historical Sociology has led to concrete methodological reflections on social and sociocultural processes, for example on the description and systematics of causes, courses and patterns (e.g., Baur 2015). In order to illustrate the conceptual foundation of PPD, selected assumptions will be connected to the framework described herein. This applies to the characteristic feature of New Historical Sociology, namely the consideration of paths and path dependencies and the fundamental consideration of events and happenings (cf. Lachmann 2013; Abbott 2001; Sewell 1996).

Assuming *path dependency* of social processes (in the sense understood here cf. Sewell 1996:262 f.) means that previous events have a formative influence on subsequent ones. The result of this precedence is that the sequence or punctuation in which action and products of action unfold has an immanent significance for the respective concrete progress of a (total) process in which certain phenomena such as groups, cultural formations, or political systems are to be located. In addition, connected to path dependency there is a strong vote to overcome “simple before‐and‐after approaches” (Schützeichel 2015:95, own translation) in favor of complex concatenations of actions, decisions, and consequences of action. While in social constructivism—chosen here as one representative of a consecutive perspective—the genesis of institutions is illustrated by rather hypothetical initial moments (Berger and Luckmann 1991:74 ff.), path dependency assumes that initial conditions of the emergence of social processes are never fully eliminated (Arrow 2004). Path dependency rests on the existence of temporal moments or periods of origins, starting points, and turning points. For the course of processes, it further implies that not all, but a multitude of events occurring over time can become process influencing (David 1985:332).

The conception of *event* (and happening as described below) as it is used here specifies the concept of path dependency. It is a necessary further addition to the social constructivist process perspective that is being undertaken here. Events represent the basic unit of the described, temporally structured paths. In the concept of “eventful temporality” Sewell (1996:262) points out: “Rather than assuming causal independence through time, it [the ‘evenemential’ conception, T. G.] assumes that events are normally ‘path dependent’, that is, that what has happened at an earlier point in time will affect the possible outcomes of a sequence of events occurring at a later point in time.” In contrast to the (nonsystematic) figure of the “pantha rhei,” this concept defines events as causers of difference, which means that the interest explicitly lies in such phenomena in time that set in motion social or cultural transformations (Sewell 1996).

This conception of eventfulness distinguishes between structure‐preserving and structure‐breaking occurrences, with “events” being “a ramified sequence of occurrences that […] is recognized as notable by contemporaries, and that […] results in a durable transformation of structures” (Sewell 2005:228). In this respect, events are in categorical contrast to *happenings* which, it is assumed, have a structure‐reproducing character (Lachmann 2013:10; Sahlins 1991). Implied here is a distinction between reproduction (routine) and transformation. Taken together, the distinction reflects a methodologically fruitful optic of discontinuity, which necessarily starts retrospectively from a break with routines (Sewell 2005:227). Events to which observers—researchers as well as field actors—ascribe eventfulness because of their consequences in the course of time are simultaneously sequenced in an uncontrolled, that is, nonlinear way.

## PROCESSUALIZING PPD: CONCEPTUAL FOUNDATIONS OF PPD AND TWO EXAMPLES

Comparing different definitions and assumptions of PPD, Baur (2011:1234 f.) argues that “process‐produced data have so far eluded methodological discussion” because, as she supposes, “basically anything human beings have left behind can be used as a social science data source.” In a critical distance to the simplistic division of “quantitative” and “qualitative data,” she argues for a much more complex classification of PPD in order to account for the multitude of this kind of data. However, despite the fact that PPDs are by definition not produced by the researcher (research elicited) and include a wide range of materials, they are inevitably part of the researcher's broader assumptions (here: with regard to the processual character of the social, Baur 2011; cf. Section “Temporality of Processes: Composing Elements of Defining PPD”). Consequentially, the question of what PPDs are, what materials they include and what they stand for refers to different *theoretical, that is, conceptual presuppositions*.

Regarding particular methods, McNeill and Chapman (2005) have argued that “[e]very research method is a means of producing knowledge, not collecting it” (p. 183). In this sense, methods are particular tools, such as optical devices, that structure empirics according to certain principles (Crotty 1998). However, methodology even more fundamentally means that research is always based on observational and research‐guiding assumptions and prior knowledge eventually shaping respective fields, expectations and accesses. In other words, methodology asks how “the inquirer [can] go about finding out whatever they believe can be known?” (Guba and Lincoln 1994:108). Quite apart from the fact that prior knowledge is always also an everyday knowledge, methodology thus accentuates that there are “general theoretical concepts” (contributing to the researcher's general definition of the object) and “object‐related concepts” (permitting the researcher to focus on particular aspects) (Kelle 2014; Meinefeld 2004:156–57).

General and object‐related concepts shape research already in early stages when certain material (and others not) is expected to be “data” that allow researchers to examine certain phenomena. These particular decisions concern New Historical Sociology in particular. Because of the analytical goal, which unites inductive and deductive logics in an independent way, theoretical and conceptual presuppositions must be disclosed in the respective research (see Lachmann 2013:10 for a similar argument). Consequently, the problematized indeterminacy, the material heterogeneity of PPD (Lachmann 2013, cf. Section “Introduction: Temporality of Processes, Process‐Produced and Digital Data”), is to be countered by an active (i.e., made explicit) conceptual predefinition that must be made transparent. Thus, process‐oriented analysis provides for the specificity of processes. The focus then is not only on general assumptions about what processes are in relation to certain social phenomena, what is it that PPD is an indication of, but even more on how these processes produce certain data; that is, how are PPD related to long‐term societal developments.

Following this demand, I suggested in Section “Temporality of Processes: Composing Elements of Defining PPD” an integration of social constructivism and path dependency, specified by the distinction on events and happenings. PPD here and hereafter are conceptualized as indications of positions of phenomena in a chronological temporal course that enable and constrain later phenomena in a particular way (paths). Two differentiating assumptions on such phenomena and its indications are applied: PPD indicate (a) the typical course of routine and its structures in their respective historical context (happening), or (b) the expression of unfolding events and potential breaks with structures (events). Such focused access to certain phenomena is to be described here on the one hand as *happening frame*, on the other hand as an *event frame* (Section “Processualizing PPD: Conceptual Foundations of PPD and Two Examples”). In order to illustrate which (different) materials are then considered PPDs and, thus, as indications for distinct types of processes, and to show the further consequences, two examples are presented here. The structure of the presentations is as follows: first, the case and research interests are briefly presented, then the concrete (empirical) specifications of PPD, and finally selected research results. A cross‐case reflection generalizes insights for the definition of PPD.

### Happening Frame

This first example comes from a sociology‐of‐knowledge research project entitled “Alfred Schütz and the Viennese Circles: Towards the communicative arrangement of incompatible knowledge,” funded by the Fritz Thyssen Foundation from 2017 to 2019. The focus of this research lies on the cultural and communicative facilitation of the production of (social) scientific knowledge in several well‐known “discussion circles” in Vienna during the so‐called interwar period, that is, after the collapse of the Habsburg monarchy at the end of World War I (1918) and before the outbreak of World War II (1939). Following the empirically stimulated thesis that it was there that different scientific positions collided in a productive way, the circles are conceptualized as “communicative knowledge cultures” (Grenz 2017; Grenz, Pfadenhauer, and Schlembach 2020). Thus, focusing on communicative knowledge cultures, the research interest mainly lies on the typical procedures and the common planning of meetings; that is, in the typical, *similarly repetitive processes of exchange*.

The research is based empirically on a comparative analysis of three different materials: First, these include ego documents that, in biographical retrospect, make the discussions in the circles and private seminars part of one's own intellectual autobiography; these include “self‐testimonies” in the form of autobiographies and other autobiographical documents. The second data form is to be emphasized here: The focus is on those documents and material expressions, which are expected to be developed in direct temporal connection with the discussions in the conversational contexts of circles and seminars. Expecting that these materials allow one to draw conclusions about the typical ways and means of communicative exchange, they were determined as PPD. These include attendance lists, preparatory notes, minutes, and diary entries made during the course of a seminar, which refer to the processes in and between particular sessions.

Drawing on this set of data, a typical course of a “private seminar” (here in reference to that of the economist Ludwig von Mises) provided an initial conversation stimulus, which was a (introductory) contribution by the mentor or a lecture by a participant. Themes were defined on the basis of an annual plan, which also provided for an overarching thematic framing valid for the respective year. Presentations comprised several sessions, insofar as, in addition to the lecture evenings, further meetings were planned for the detailed discussion of the presentations. Considerable importance was given to the discussion following a presentation. Process‐produced documents refer to a meticulous preparation of the contributions to these discussions. The seminar participants wrote pages long, detailed documents with titles such as “Guidelines for Discussion,” “Discussion Notes,” or “Discussion Speech.” The duration of the discussion also followed a predefined time structure (of about two hours), which required a change of location. It opened a specific “space” for the continuation of an in‐depth theoretical discussion, which was not abandoned in the course of the evening but continued less strictly. After an equally institutionalized period, there was typically another change of location.

### Event Frame

The second frame is illustrated by research that has been conducted on a much more recent subject. In particular, the following takes up research insights and follow‐up work from the project “Mediatization as a Business Model: The Entanglement of Product and Adoption in a Digital Media Environment and its Consequences,” a subproject of the interdisciplinary priority program “Mediatized Worlds,” funded by the German Research Foundation (DFG) between 2012 and 2014. This project—and several later, follow‐up investigations of the author (see Grenz and Eisewicht 2017; Grenz and Kirschner 2018)—aimed at the empirical and theoretical deepening of unforeseen and unintended consequences of mediatization. Starting with an interest in the dynamics of Online Poker and Application Stores (Grenz 2013), the focus was on how and to what extent media‐induced pushes and jumps in social worlds are based to a considerable extent on unintended effects. According to the research‐led thesis and assumptions, these effects are triggered by developers and providers when they weave digital technologies into fields of action. The focus of interest was on how media technologies are appropriated, whether, when and how the companies that developed and offered them “react” to problematic appropriations—that is, how these appropriations affect corporate and product strategies. The histories of today's sociotechnical fields are characterized by frictional negotiations which *unfold in a complex way at certain times or over certain periods* and involve different groups of actors. Some of these negotiations result in significant structural breaks regarding the regulations and the technical forms, such that they turn out to be *events* as described above.

This research is therefore particularly interested in events, how they unfold over time and what consequences they have. Consequently, particular “sources” of material come into view: In addition to interviews and retrospective presentations, the materials that are decisive here are those which the researcher expects to express past activities and interactivities, but which are not based on actors’ explicit reconstructions of past events. PPD here are characterized by the fact that they are generated at certain points in time. Thus, their exact timing in a chain of (inter)actions is elementary for the reconstruction of the event's temporal unfolding. This includes, for example, chronological blog entries, press releases, instructions for developers, and also the journalistic contributions that represent the respective developments at the time of the event. Research in and about today's modalities of the Internet makes it possible to record the respective time of origin for almost every date and thus to classify it in the time horizon of a temporal trajectory. On the other hand, Internet‐based research is not only able to provide insights into the “here and now,” but also a systematic “going back into the past” (Baumgarten and Grauel 2009:100). Projects such as the “Internet Archive” (cf. the “Wayback Machine”) allow researchers to access later modified, postponed, or even deleted website versions, blog entries, messages, and file versions—and this to the day.

Oriented toward the event frame, the author has conducted several trajectory studies, for example, on the unfolding dynamics of the so‐called “Cambridge Analytica Scandal” (which, starting from 2014, consolidated Facebook's data monopoly and at the same time triggered significant changes in algorithmic interfaces) and the so‐called “In‐App‐Purchase” hack (which led to the development of a new type of payment security in 2012 and later to the reintroduction of methods in order to identify unique users; Grenz and Kirschner 2018:622). Insights from case studies and their comparison suggest that these current fields, due to their inherent frictional negotiation processes, can be interpreted as arenas. Here, different social worlds overlap in a conflicting way, insofar as different objectives and plans of action of the respective core actors (entrepreneurs, developers, outlaws, users, etc.) collide. Thus, arenas unfold in an interplay of differently motivated actors which is sociotechnically mediated. The (interim) results affect millions of people. Respective negotiations concern questions of what is regarded as legitimate action, or legitimate use of digital media technology. Focusing on the temporal unfolding of events in this way provides sociology of the digital with a methodological framework—an exclusive access to the contemporary emergence of sociotechnical order (Grenz and Eisewicht 2017:11; Lash 2003:54).

Summarizing the role of PPD in view of both presented strands of research, two different determinations can be identified: PPD in the first frame (a) is expected to pertain to typically repeated sequences of (more or less actively planned) action. It is of crucial importance that data here are supposed to be generated “en passent” to daily routine action, like traces in the manner set out in an earlier section. PPD in the second variant (b) are considered in regard to a different temporal relevance, its chronologic signature. The researcher expects them to be part of an unfolding, temporally punctuated process over time. Thus, it is not relevant that material has been generated by actors without communicative intention.

However, in both frames and the corresponding examples given it is possible to go beyond the respective process reconstruction and ask for a possible linkage with more long‐term processes. Respective results of the process‐oriented analysis thus are *supplemented* to the indication of overall societal changes. There are different conceptions of those overarching processes researchers can apply (and have to justify), two shall be described here: for one thing, broader sociohistorical contexts, for another, “metaprocesses” (Krotz 2007:257; van der Loo and van Reijen 1992). In recent years, the examination of metaprocesses has been particularly promoted in the Sociology of Media and Communication, especially in regard to “mediatization” as a long‐term metaprocess (Krotz 2008; Krotz and Hepp 2011). By making significantly more general connections to sociohistorical contexts, certain relations of micro‐, meso‐, and macrolevels can be put forward in a specific way. Further, drawing on “metaprocesses,” that is, allocating certain insights in a more abstract way, renders it possible to assign and structure different (sub)processes to particular overarching—empirically not graspable—metaprocesses. In doing so, metaprocesses serve as more abstract forms of “conceptual constructs” (cf. Section “Processualizing PPD: Conceptual Foundations of PPD and Two Examples”), namely those that are intended to make long‐term historical change describable by assigning various subprocesses and phenomena to a wider “panorama” (Hepp 2013:68). In order to exemplify these differences, the exemplary cases again will be used.

The analyzed process in the case of the historical discussion circles (a) can initially be located *sociohistorically*. Thus, it falls into a historical period of considerable social upheaval which, as a result of World War I, occurred not only for universities and academics. The upheaval led, among others, to the marginalization of social groups and their retreat, and non‐university circles became the starting point for the development of a distinctive “interpretative” social research (Pfadenhauer and Grenz 2020:10). The contested digital infrastructures (b) can be assigned to a wider *metaprocess*. For example, the theory of “Reflexive Modernization” presupposes that nonintended side effects of political, economic, and technological progress confronts society with its own developments, eventually leading to a nonlinear, discontinual transformation (Beck, Giddens, and Lash 1994; Lash 2003). Societal actors (as companies and developers) answer on the side effects by “quick decisions,” which, with regard to their response speed, are almost reflex like; this “logic” becomes the symptomatology for an action that (still) has no adequate solutions to new problems (Lash 2003:51). In this respect, the above presented process insights are used as a microfoundation of a metaprocess—“Reflexive Mediatization” (Grenz 2013; see Grenz, Möll, and Reichertz 2014)—that emphasizes the nonintended side effects of progressive digitalization.

## CONCLUSION AND OUTLOOK

The aim of this article was to take up the repeatedly stressed ambivalence of PPD and the respective need for further methodological reflection. It has been argued that particular compositions of general and object‐related conceptions have to be made transparent (Meinefeld 2004) and, consequentially, persuasive in a “rhetorically‐performative” way (Eisewicht and Grenz 2018:371, own translation). That is to say, that and how theoretical assumptions and respective decisions do not only become relevant within preparation, analysis, and evaluation of data, but already with the basic conceptions of processes, their temporality, and consequently the definition of data. PPD thus prove to be data that are indicators for processes, which in turn are determined in advance by the researcher. Thus, there can be no empirics or empirical research without theory, and this also applies to the question of what constitutes data (PPD). This is what distinguishes methodology at its core.

As shown, because of the material heterogeneity and plurality of PPD this reflection is of particular importance. But its importance also stems from another fact. Similar to the notion of “pure” data as it was claimed by quantitative diachronic social science (cf. Section “Introduction: Temporality of Processes, Process‐Produced and Digital Data”), currently there is a peculiar restrengthening of the taken‐for‐grantedness of data and its evidence character—especially in the course of so‐called “digital sociology”—which would render any theoretical reflection unnecessary (Anderson 2008; Mosco 2014; regarding the debate Hepp et al. 2018). Starting from social constructivism, extended by New Historical Sociology, the argumentation and determination of PPD undertaken here thus also contributes to this core challenge for the “sociology of the digital” (not digital sociology). By transferring for the first time core concepts of the New Historical Sociology to the sociology of the digital, the potentials of a methodologically founded, process‐oriented access to the emergence of digital order become apparent.

However, it must be stressed that making conceptual frames and prior assumptions transparent—as it is the case with the difference of “events” and “happenings” (see above)—this does not imply a predetermination of the research process, or research outcomes. That is, events, for one thing, always consist of cascades of effects and, at the same time, social attributions by different actors (Gilmore and O'Donoughue 2015:5; Sewell 1996:843, 2005:228). Thus, diachronic research as defined here, no matter what concrete approach it takes, should take serious interpretations of field actors. It should at least not dismiss them hastily in favor of other data (Bowen 2006). Furthermore, the researcher must be able to be irritated by PPD and, consequently, pursue inductive sampling strategies (see, especially Charmaz 2006:96 ff.) in order to compose respective processes. In this regard, it should be emphasized that various aspects relating to PPD have been deliberately omitted in this article. This mainly concerns the interpretation of PPD, with which, depending on the process concept and the targeted insights, different procedures, and combinations of procedures are linked.
